# Regulation of Monospecies and Mixed Biofilms Formation of Skin *Staphylococcus aureus* and *Cutibacterium acnes* by Human Natriuretic Peptides

**DOI:** 10.3389/fmicb.2018.02912

**Published:** 2018-12-10

**Authors:** Andrei Vladislavovich Gannesen, Olivier Lesouhaitier, Pierre-Jean Racine, Magalie Barreau, Alexander I. Netrusov, Vladimir K. Plakunov, Marc G. J. Feuilloley

**Affiliations:** ^1^Department of Microbiology, Faculty of Biology, Lomonosov Moscow State University, Moscow, Russia; ^2^Laboratory of Petroleum Microbiology, Winogradsky Institute of Microbiology, Research Center of Biotechnology, Russian Academy of Sciences, Moscow, Russia; ^3^Laboratory of Microbiology Signals and Microenvironment, EA4312, University of Rouen Normandy, Évreux, France

**Keywords:** *Staphylococcus aureus*, *Cutibacterium acnes*, multispecies biofilms, natriuretic peptides, temperature dependent regulation, human skin–microbiome interactions

## Abstract

*Staphylococcus aureus* and *Cutibacterium acnes* are common representatives of the human skin microbiome. However, when these bacteria are organized in biofilm, they could be involved in several skin disorders such as acne or psoriasis. They inhabit in hollows of hair follicles and skin glands, where they form biofilms. There, they are continuously exposed to human hormones, including human natriuretic peptides (NUPs). We first observed that the atrial natriuretic peptide (ANP) and the C-type natriuretic peptide (CNP) have a strong effect *S. aureus* and *C. acnes* biofilm formation on the skin. These effects are significantly dependent on the aero-anaerobic conditions and temperature. We also show that both ANP and CNP increased competitive advantages of *C. acnes* toward *S. aureus* in mixed biofilm. Because of their temperature-dependent effects, NUPs appear to act as a thermostat, allowing the skin to modulate bacterial development in normal and inflammatory conditions. This is an important step toward understanding how human neuroendocrine systems can regulate the cutaneous microbial community and should be important for applications in fundamental sciences, medicine, dermatology, and cosmetology.

## Introduction

Skin microorganisms that compose the skin microbiome form as a complex community in which all members are closely interrelated (Belkaid and Hand, 2014; Prescott et al., 2017). This community evolved with humans and became an essential part of the human body (Chiller et al., 2001). *Staphylococcus aureus* and *Cutibacterium acnes* are main actors in the human skin microbiome and prefer sebum-rich microniches, such as skin gland hollows and hair follicles (Grice et al., 2009; Aubin et al., 2014; SanMiguel and Grice, 2015; Feuerstein et al., 2017). Both *S. aureus* and *C. acnes* are opportunistic pathogens, and can sometimes be at the origin of skin disorders. In this way, *C. acnes* is considered to be one of the causes of acne vulgaris (Fitz-Gibbon et al., 2013; Achermann et al., 2014; Petersson et al., 2018). In parallel, it has been shown that *C. acnes* can be a cause of complications after operations and the setup of prosthetic implants (Howlin et al., 2017). Thus far, the role of *S. aureus* in acne vulgaris remains unclear (Khorvash et al., 2012; Totté et al., 2016). However, it was shown that colonization of acne lesions by *S. aureus* can strengthen inflammation (Totté et al., 2016; Dreno et al., 2017). Moreover, *S. aureus* can be responsible for complications and inflammation during psoriasis and skin wounds (Elfatoiki et al., 2016; Lacey et al., 2016; Totté et al., 2016). Both bacteria are able to form biofilms inside the niches that they colonize (Daum, 2007; Jahns et al., 2012; Jahns and Alexeyev, 2014). Biofilms are microbial communities embedded in an extracellular polymeric matrix, conferring on bacterial cells included in these structures protection against unfavorable environmental factors (Nikolaev and Plakunov, 2007). Multispecies biofilms are the most common form of microbial life on Earth (Nozhevnikova et al., 2015), and human skin is not an exception. It is likely that multispecies microbial biofilms, including those formed by both *C. acnes* and *S. aureus*, can be a cause of acne vulgaris and other skin disorders, because such biofilms are formed inside comedones and hair follicles (Ten Broeke-Smits et al., 2010; Jahns et al., 2012; Khorvash et al., 2012; Matard et al., 2013; Jahns and Alexeyev, 2014).

There is much evidence that the microbial community of skin is not just closely related with the human organism, but exchanges information with its host. Those connections seem to be at least partly dependent on molecules that are not classically considered as bacterial effectors, such as hormones, neuropeptides and neurotransmitters. For instance, it was shown that *Escherichia coli* possess several adrenaline receptor/sensors. Those catecholamines (adrenaline and noradrenaline) are able to regulate a number of processes in bacteria such as toxin synthesis, growth, and pili synthesis (Hughes and Sperandio, 2008). Near neurotransmitters, peptidic hormones are also able to modulate bacterial physiology (Lesouhaitier et al., 2018). In this way, one of the most abundant neuropeptides of skin – substance P, which is released by skin nerve endings and diffuses in sweat and skin – exerts a central role in expressing virulence in cutaneous strains of *S. aureus* and *Staphylococcus epidermidis* (Feuilloley, 2018 for review). In *S. aureus*, substance P increases staphylococcal enterotoxin C2 synthesis and *S. epidermidis* biofilm formation activity (N’Diaye et al., 2016). Conversely, at high concentrations, substance P and many other neuropeptides, such as neuropeptide Y, can exert antimicrobial activities against different bacterial species including *E. coli, Enterococcus faecalis*, or *Lactobacillus acidophilus* (El Karim et al., 2008).

Recently, natriuretic peptides (NUP) were of special interest, specifically in the field of microbial endocrinology as described by Lyte (2004). To date, NUPs are represented by three main members: atrial natriuretic peptide (ANP), brain natriuretic peptide (BNP), and C-type natriuretic peptide (CNP) (Potter et al., 2009). Among these peptides, it has been observed that CNP is more active on *Pseudomonas aeruginosa*. CNP was shown to modulate toxins and lipopolysaccharide synthesis in *P. aeruginosa* (Veron et al., 2007; Blier et al., 2011). It was observed that CNP increases *P. aeruginosa* and *P. fluorescens* cytotoxicity (Veron et al., 2007, 2008) as well as *P. aeruginosa* virulence in a *Caenorhabditis elegans* model (Blier et al., 2011). On the other hand, it was demonstrated that CNP strongly decreases *P. aeruginosa* biofilm formation and that these effects are specific and consecutive to the binding of this peptide on a bacterial sensor named AmiC, which is an ortholog of human C-type NUP receptors (Rosay et al., 2015). NUPs are synthesized by different human cells, including heart (atrium) cardiomyocytes and the endothelial cells of blood vessels. They are transported by the blood and produced locally by the capillary walls, and could impact the human skin microbiota in areas particularly rich in capillary vessels. In this regard, the bulge of the hair follicle is a region of special interest because of the presence of a dense network of capillary vessels in the immediate vicinity of the hair follicle hollow (Xiao et al., 2013) where gland ducts open to a rich microbial community, including *C. acnes* and *S. aureus* (Lange-Asschenfeldt et al., 2011; Matard et al., 2013). So, these two bacteria in this area are under permanent exposure to NUPs, which can potentially affect bacterial growth and biofilm formation as well as, through this, general skin status. Recently we have shown that ANPs and CNPs affect the growth of binary biofilms of *S. aureus* and *S. epidermidis* (Gannesen et al., 2018b). This work aims to investigate the impact of ANPs and CNPs on monospecies and binary biofilms of *S. aureus* and *C. acnes*. This should enlarge our understanding of skin–microbiome interactions.

## Materials and Methods

### Strains and Cultivation

*Staphylococcus aureus* MFP03 was isolated from the skin of a healthy volunteer and was fully characterized (Hillion et al., 2013). The strain was stored at -140°C in a cryofreezer. For the bacterial culture, an aliquot of bacteria was mixed in 25 ml of Luria Bertani medium (LB) and incubated for 24 h at 37°C under agitation.

*Cutibacterium* (former *Propionibacterium*, Scholz and Kilian, 2016) *acnes* strains ribotype 4 (RT4) HL045PA1 and ribotype 5 (RT5) HL043PA2 (acneic strains), initially isolated by Fitz-Gibbon et al. (2013), were obtained from BEI Resources American Type Culture Collection (VA, United States). These strains are associated with severe forms of acne and differ from non-acneic strains (ribotype 6 and in a lower extend ribotypes 1, 2, and 3) by a large plasmide (Locus 3), which should confer their virulence properties (Fitz-Gibbon et al., 2013). Bacteria stored at -140°C were initially plated on Reinforced Clostridial Medium (RCM) agar. As these strains are strictly anaerobic, the plates were incubated for 72 h in a BD GasPack^TM^ under anoxic conditions at 37°C. Colonies were then transferred into sterile conical 50 mL tubes (Falcon) filled to maximal capacity with RCM and grown for 72 h at 37°C.

### Natriuretic Peptides

Human ANP (Alfa Aesar, United States) has a molecular weight of 3080.47 g/mol and CNP (PolyPeptide, Strasbourg, France) has a molecular weight of 2196.1 g/mol. Peptides were reconstituted in milli-Q water and stored dissolved at -20°C with molar concentration 1.623 × 10^-4^ M and 4.554 × 10^-4^ M for ANP and CNP, respectively. According to data in the literature on the physiological NUP concentrations in humans (Edwards et al., 1988; Stingo et al., 1992) and data of NUP concentrations used in experiments with eukaryotic cells (Klinger et al., 2013), we studied the effect of the peptides at concentrations ranging from 10^-6^ to 10^-8^ M, as well as with a mix of the two peptides at a concentration of 10^-6^ M. Peptides were added to experimental medium immediately before the experiment’s start.

### Bacterial Monospecies Cultures Growth Dynamics

The effects of ANP and CNP on monospecies culture growth were studied in a Bioscreen C system (Finland) using 100-well flat-bottom Honeycomb microtiter plates (Growth Curves, United States). On the bottom of the well, an aliquot of NUP was distributed. Then 200 μl of growth medium were injected in each well. *S. aureus* MFP03 was cultivated in tryptic soy broth (TSB, Sigma) with addition of 0.25% glucose (Sigma). *C. acnes* strains were cultivated in RCM medium. Before plating, bacterial cultures were washed twice with physiological saline (PS) at pH 7.0. Then OD_580_ of cell suspension in PS was adjusted to 1.0. Finally, 16 μl of culture were injected into each well containing medium and peptides. Some wells were maintained without a culture medium (negative control) and some others without peptides (positive control). *S. aureus* MFP03 was cultivated aerobically at 37 or 33°C (closer to the skin’s physiological temperature). *C. acnes* was also cultivated at 33 and 37°C as acneic strains naturally develop in anoxic deep skin regions. In the case of *C. acnes*, peripheral wells were filled with a CO_2_-producing solution and prepared in anoxic conditions using a GasPack^TM^ system and sealed with parafilm before incubation. The optical density of the cultures was determined automatically every 15 min. Growth curves were determined over a minimum of three independent experiments.

### Confocal Laser Scanning Microscopy of Monospecies Biofilms

Monospecies biofilms of *S. aureus* MFP03 and *C. acnes* RT5 were obtained in 24-well plates with flat glass bottoms (Greiner Bio-One, Germany). Biofilms were grown according to Gannesen et al. (2018a,b). Briefly, prepared cultures were washed twice with sterile PS and OD_580_ was adjusted to 1.0. In each well 300 μl of cell suspension were added, and plates were incubated 2 h at room temperature (time for cell adhesion). *C. acnes* strains were incubated in a GasPack^®^ (BD) system in an anaerobic atmosphere. In each well the suspension was removed, and wells were washed twice with sterile PS to remove non-attached cells. One well was dried and fixed in order to verify adhesion control. One milliliter of medium containing ANP or CNP, or no peptides (positive control), was injected into the wells. To find out the probable effect of cultivation conditions on biofilm growth, different conditions for each bacterium were tested. They are summarized in Table 1.

**Table 1:** Conditions of monospecies biofilm cultivation for CLSM analysis.

Strain	*S. aureus* MFP03				*C. acnes* RT4, RT5	
Conditions	37°C, aerobic atmosphere, TSB	33°C, aerobic atmosphere, TSB	33°C, anaerobic atmosphere, TSB	33°C, anaerobic atmosphere, RCM	37°C, anaerobic atmosphere, RCM	33°C, anaerobic atmosphere, RCM

*TSB, trypticase soy broth; RCM, reinforced clostridial medium.*

Biofilms were grown for 24 h for *S. aureus* MFP03 and for 72 h for *C. acnes*. In each case, where anaerobic atmosphere was necessary, a GasPack^®^ system (BD) was used. After incubation time, biofilms were washed twice with sterile PS and stained with SYTO 9^®^ Green fluorescent dye for 20 min. The staining dye was then removed and the biofilms were rinsed and fixed with ProLong^®^ Diamond Antifade Mountant (Molecular Probes^TM^). Observations were realized using an LSM 710 inverted confocal laser-scanning microscope (Zeiss, Germany). Three-dimensional (3D) images and orthocuts were obtained using Zen^®^ 2009 software. Biofilm thickness was quantified with the same software. Images are representative of the biofilm structure observed in a mean of 20 different fields over a minimum of four independent studies. Biofilm density and thickness were calculated over the same number of observations using Zeiss Zen Image Analysis software for light microscopy (ImageJ software package). Statistical differences were determined using the Mann–Whitney non-parametric test. The differences between the experimental and control variants were considered reliable at a confidence coefficient >95% (*P* < 0.05).

### Study of Bacteria Interaction in Binary Biofilms

Binary biofilms were obtained on Petri dishes with RCM agar. Whatman GF/F glass fiber filters 21 mm in diameter were used as carriers. Cultures were prepared as described above. Sterile filters were placed onto the RCM surface, after which 20 μl of each culture were dropped onto filter center. Biofilms were incubated for 72 h in an anaerobic atmosphere at 37 or 33°C. At 33°C both *C. acnes* RT5 and *S. aureus* MFP03 were used, and different types of biofilms were constructed and are described in Table 2. Before plating the secondary colonizers, all filters with biofilms were replaced on Petri dishes with fresh RCM agar. Secondary colonizers were plated onto the biofilm as a drop of a culture prepared as described above.

**Table 2:** Types of binary biofilms of *S. aureus* MFP03 and *C. acnes* RT5 studied at 33°C.

Component Binary biofilm type	Pre-formed biofilm	Secondary colonizer
Simultaneously growing	None	None
Based on aerobically pre-formed biofilm of *S. aureus* MFP03	24 h biofilm of *S. aureus* MFP03, formed under aerobic atmosphere	*C. acnes* RT5, growth for 72 h under anaerobic atmosphere
Based on anaerobically pre-formed biofilm of *S. aureus* MFP03	24 h biofilm of *S. aureus* MFP03, formed under anaerobic atmosphere	*C. acnes* RT5, growth for 72 h under anaerobic atmosphere
Based on pre-formed biofilm of *C. acnes* RT5	72 h *C. acnes* RT5 biofilm, formed under anaerobic atmosphere	*S. aureus* MFP03, growth for 24 h under anaerobic atmosphere

In parallel, as control conditions monospecies biofilms were grown and manipulated in the same way as binary biofilms. All samples were divided into two parts: one was dedicated to metabolic activity analysis of the cells, while the second one was used for *S. aureus* MFP03 counting. Experiments were conducted independently at least three times. Statistical differences were determined using the Mann–Whitney non-parametric test. The differences between the experimental and control variants were considered reliable at a confidence coefficient >95% (*P* < 0.05).

### Determination of Cells’ Metabolic Activity in Biofilms

The metabolic activity of cells in biofilms was determined as described by Gannesen et al. (2018a,b), using the method of Plakunov et al. (2016). Briefly, filters with biofilms were stained with 3-(4,5-dimethyl-2-thiazolyl)-2,5-diphenyl-2H-tetrazolium bromide (MTT). MTT is an acceptor of electrons from electron-transport pathways; when reduced, it becomes formazan which is non-soluble in water. The amount of formazan generated is proportional to the metabolic activity of the bacteria. Filters with biofilms were stained with a 0.2% solution of MTT in sterile LB for 30 min. Later, filters were washed with distilled water in order to remove MTT, then dried, and formazan was extracted with dymethylsulfoxide (DMSO, Sigma). Subsequently, the optical density of extracts was measured at OD590.

### *S. aureus* MFP03 CFU Count in Binary Biofilms

After plating the biofilms, the amount of CFU in 20 μl of the initial culture was determined by plating on Petri dishes for control. After the maturation of biofilms, i.e., after 24 or 72 h of culture, filters were homogenized in 5 ml of sterile PS: first by stirring with a glass rod, followed by vortexing for 30 s. Glass fibers played the role of an additional abrasive material for the disintegration of cell aggregates, allowing for maximal recovery of the bacteria (tests for *S. aureus* biofilm dispersal and the role of glass fibers as abrasive agent were conducted. Data not shown). After biofilm dispersal, cell suspensions were diluted and aliquots were plated on tryptic soy agar (TSA, Sigma) Petri dishes supplemented with 0.25% glucose. Petri plates were incubated at 37°C for 48 h, and CFU numbers were counted. Experiments were repeated independently at least three times.

### Impact of ANP and CNP on Binary Biofilms of *S. aureus* MFP03 and *C. acnes* RT5

The general principle of the determination of the effects of ANP and CNP were performed as described in Gannesen et al. (2018a,b). Briefly, biofilms were grown on glass fiber filters Whatman GF/F in RCM in 12-well plates (Thermo Fisher Scientific, United States). One filter was placed on the bottom of the wells, where 3 ml of RCM containing NUP were previously added. Only concentrations of NUPs with the strongest effect on monospecies biofilms found in CLSM experiments were studied. Samples without NUPs constituted positive controls. Subsequently, 50 μl of cell cultures, prepared as described above, were added. In parallel, monospecies cultures of biofilms for each bacterium were cultivated in the same conditions as the controls. Biofilms were incubated at 33°C for 72 h under anaerobic atmosphere (GasPack^®^). All samples in each experiment were duplicated on two filters; after incubation one filter of each sample type was stained with MTT and one was processed to count CFU. Experiments were conducted at least in triplicate.

### Fluorescence *in situ* Hybridization (FISH) of *S. aureus* MFP03 and *C. acnes* RT5 in Binary Biofilms

Biofilms were grown in 24-well plates with a flat glass bottom (Greiner Bio-One, Germany). Three types of binary biofilms were constructed and studied (Table 3).

**Table 3:** Variants of binary biofilms for FISH-labeling and CLSM analysis.

Component Binary biofilm type	Pre-formed biofilm	Secondary colonizer
Simultaneously grown	None	None
Aerobically pre-formed *S. aureus* MFP03	Biofilms of *S. aureus* MFP03, formed for 24 h under aerobic atmosphere in presence of NUPs	*C. acnes* RT5, which was plated on 24 h biofilm of *S. aureus* MFP03. Biofilms were incubated for 72 h in presence of NUPs under anaerobic atmosphere.
Pre-formed *P. acnes*	Biofilms of *C. acnes* RT5, formed for 72 h in presence of NUPs	*S. aureus* MFP03, which was plated on 72 h biofilm of *C. acnes* RT5. Biofilms were incubated for 24 h in presence of NUPs under anaerobic atmosphere.

In case of simultaneously grown biofilms and primary colonizers in pre-formed biofilms, biofilms were obtained as described above. For simultaneously grown biofilms, 300 μl of each culture per well were injected. Simultaneously, grown biofilms were cultivated for 72 h. All biofilms were incubated in an anaerobic atmosphere at 33°C in RCM without agitation. In case of pre-formed biofilms, when the incubation time of the primary colonizer was over, biofilms were washed twice with sterile PS. Then, 1 ml of fresh medium with ANP or CNP was added in each well, and 40 μl of culture of the secondary colonizer were added. As in previously described experiments, we studied the most effective concentrations of NUPs found by CLSM in monospecies biofilms. Then binary biofilms were incubated for the necessary time. In parallel, controls for hybridization were made by preparing monospecies biofilms of each bacteria by the same pathway as binary ones. The positive control was without the addition of NUPs. All experiments were made independently in triplicates.

FISH-labeling was performed as described by Nistico et al. (2014). Briefly, when the incubation time was over, biofilms in wells were washed twice with sterile PS and fixed with 100% methanol for 30 min at room temperature. Subsequently, methanol was removed, and biofilms were dried overnight at room temperature. Fixed biofilms were treated with a lysozyme (Sigma) solution (0.1 mg/ml in buffer 0.1 M Tris–HCl pH 8.0 + 0.05 M EDTA) at 200 μL per well. A piece of paper towel soaked with water was placed in a hermetically sealed bag from the GasPack^®^ system to maintain high humidity and closed plates with biofilms were introduced inside. Lysozyme treatment was conducted at 37°C for 3 h. Subsequently, the lysozyme solution was removed gently by pipetting, and 200 μl of the lysostaphin solution [Sigma; 10 mg/ml in Tris–HCl pH 8.0 + 0.01% of sodium dodecylsulfate (SDS) + 20% formamide] was added in each well. Plates were incubated in the same system for 10 min at 37°C. Then, the lysostaphin solution was removed, and wells were washed twice with a phosphate buffer (pH 7.4). Cell membranes were permeabilized by exposing them for 3 min to increasing concentrations of ethanol (50, 80, and 100%) in the pH 7.4 phosphate buffer at room temperature. After treatment, the wells with biofilms were properly dried at room temperature.

For *C. acnes* RT5 hybridization, a 5′-GCCCCAAGATTACACTTCCG-3′ (Eurogentec) probe was used, as described by Poppert et al. (2010). On 5′ the probe was marked with the fluorescent dye Alexa Fluor 546. The probe was dissolved in 50 μl of sterile milli-Q water and stored at -20°C before use. For experiments, a hybridization buffer was prepared in a 2-ml Eppendorf tube by mixing 360 μl of 5 M NaCl in 40 μl of 1 M Tris–HCl (pH 8.0) and 600 μl of formamide (30% of volume). The solution volume was adjusted to 2 ml with milli-Q water, and 2 μl of 10% SDS were added. Tubes with buffer were enveloped in aluminum foil to avoid light flashing. Directly before experiments, 1.5 μl of probe solution was added into the buffer so that the final concentration of the probe reached 50 ηg/μl. In each well with biofilms, 200 μl of hybridization mix were added. Plates were quickly closed and placed into GasPack^®^ hermetic bags with wet paper towels inside; bags were closed and immediately enveloped in aluminum foil to avoid any light. Hybridization was conducted for 1 h at 46°C. While this occurred, a washing buffer was prepared. In conical 50 ml centrifuge tubes (Corning), 1 ml of 1 M Tris–HCl (pH 8.0), 1.02 μl of 5 M NaCl and 500 μl of 0.5 M sodium ethylene diamine tetraacetate (EDTA) were mixed. The volume was then adjusted to 50 ml with sterile milli-Q water, and 50 μl of 10% SDS were added. After hybridization, the liquid was removed from the wells, 1 ml of washing buffer was added in each well, and plates were incubated in darkness at 48°C for 15 min. Then, the liquid was removed and each well was rinsed with milli-Q water, then dried at room temperature in darkness. Afterward, 100 μl of DAPI-containing ProLong Diamond Antifade Mountant (Thermo Fisher Scientific) were added in each well to fix the biomass and stain it with DAPI. Plates were covered with aluminum foil and incubated at 4°C overnight.

CLSM analysis was conducted in the red and blue modes of fluorescence. For Alexa Fluor 546, the laser line 488 nm was used. DAPI was used as total biomass of *S. aureus* MFP03 and *C. acnes* RT5 indicator. For every sample, analysis was realized over at least 20 observations and by scanning at least 5 of the most representative areas. LMSM 710 was upgraded with a 405 nm laser diode, allowing the visualization of the DAPI staining, and scanning was performed in the red, blue, and simultaneously red-blue modes. The biomass density of *C. acnes* RT5 and *S. aureus* MFP03 biofilms was studied and calculated using the Comstat 2 plug-in of ImageJ to determine the variations of *S. aureus* and *C. acnes* biomass after exposure to ANP or CNP.

### Protein-BLAST Search

According to Rosay et al. (2015), the amidase operon regulator AmiC of *P. aeruginosa* has been identified as the receptor/sensor for CNP. We searched for proteins in *Propionibacteriaceae* and *Staphylococcaceae* taxon representatives, which could be similar to AmiC of *P. aeruginosa*. After this, the search for *C. acnes* amidase homologies in taxons *Pseudomonas, P. aeruginosa*, and *Staphylococcaceae* was conducted. A protein-BLAST search was made on the NCBI website of the National Institutes of Health in the United States^1^ .

### Statistical Analysis

All experiments were conducted in at least three independent repeats. The statistical significance of results was evaluated using the non-parametric Mann–Whitney test. A significant difference was marked with ^∗^ for *p*-value <0.05.

## Results

### Growth Dynamics of *S. aureus* MFP03 and *C. acnes* Strains in the Presence of NUPs

**FIGURE 1:**
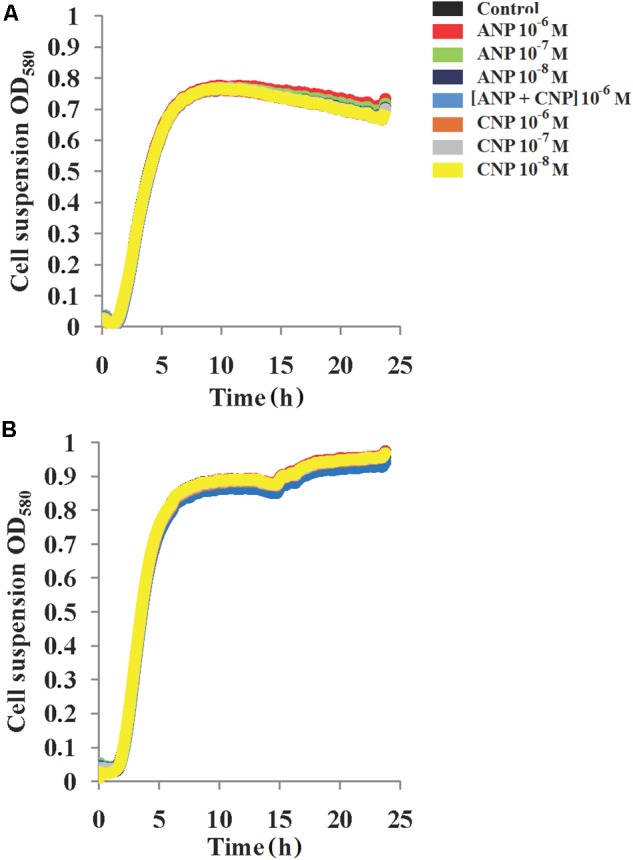
Growth dynamics of *S. aureus* MFP03 monospecies cultures in the presence of ANP and CNP at different temperatures **(A)** 37°C and **(B)** 33°C.

At 37°C (Figure 1A), the growth of *S. aureus* MFP03 (0.6 h^-1^), generation time (61 min) and death constant after stationary phase 0.009 h^-1^ were measured. The maximal OD_580_ in control conditions reached 0.8 and the mean lag-phase length was about 1 h. Adding ANP or CNP 10^-8^ to 10^-6^ M did not significantly affect any parameter of *S. aureus* MFP03 growth. The same observations were made using ANP and CNP (10^-6^ M each) in association. At 33°C, the growth curves of *S. aureus* MFP03 were slightly different in comparison with ones at 37°C (Figure 1B). The growth constant was higher (0.63 h^-1^), and the generation time was slightly increased (63 min), but cells did not enter into a death phase characterized by reduction of OD, maybe because they grew slower (lag-phase 1.5 h) and with higher maximal OD_580_ (about 0.97). As observed at 37°C, addition of NUPs did not significantly modify *S. aureus* growth parameters.

**FIGURE 2:**
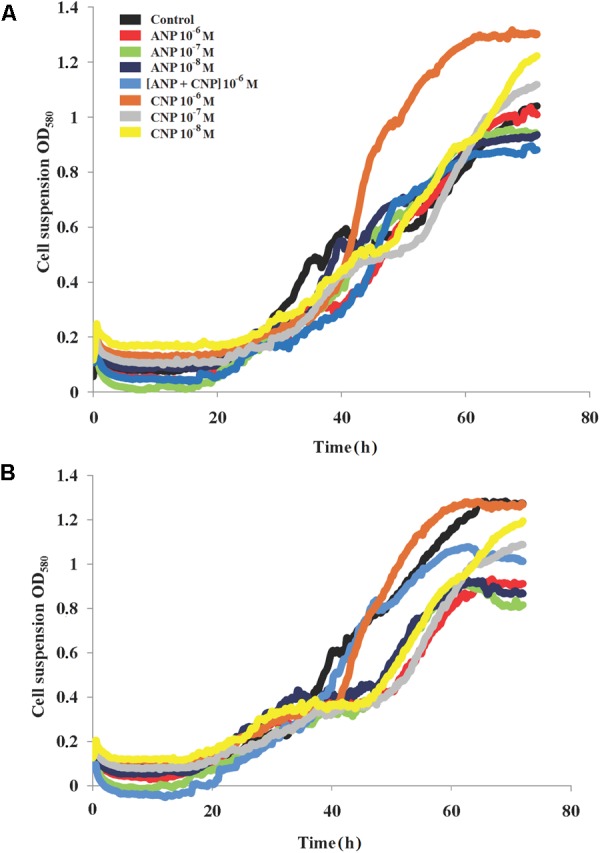
Growth dynamics of *C. acnes* RT4 and RT5 monospecies cultures at 37°C in the presence of ANP and CNP. **(A)** *C. acnes* RT4 and **(B)** *C. acnes* RT5.

In contrast, we observed that the growth of *C. acnes* RT4 and RT5 at 37°C was impacted by NUP exposure (Figure 2). More precisely, in control conditions the strain RT5 showed a growth constant of 0.12 h^-1^, a generation time of 7 h and a death rate constant at 0.0003 h^-1^. Adding ANP and CNP markedly increased the generation time of this bacterium. The maximum reached +112.4 and +60.5% for ANP and CNP, respectively. However, the association of the two NUPs only increased the generation time of +15.7%, and no additive or synergistic effect of combining ANP and CNP was observed. In the presence of both ANP and CNP the growth constant varied from 0.05 to 0.08 h^-1^.

Concerning death rate constants, no variation was observed for any NUP concentration that was tested. For the strain RT4 in the control condition, we measured a generation time of 7.8 h, a growth constant of 0.09 h^-1^ and a death rate constant at 0.015 h^-1^. The addition of both peptides increased the generation time to 10.28 h (ANP 10^-6^ M) or 12.25 h (CNP 10^-8^ M) (Figure 2A). When the bacteria were exposed to a combination of the two NUPs, no additional or synergistic effect was observed. The growth constant in the presence of NUPs was slightly decreased, varying from 0.06 to 0.08 h^-1^. This was probably because of the reduction in growth rate, however, the death rate was constant and varied near zero. The RT4 strain’s maximum OD increased in the presence of CNP and slightly decreased in presence of ANP. The same was observed with the RT5 (Figure 2B) strain in the presence of CNP 10^-6^ M. In the case of *C. acnes* it appeared that both NUPs were capable of modulating the growth dynamics, although these effects were generally moderate. At 33°C, the effects of both NUPs were virtually as marginal as observed at 37°C (data not shown).

### Confocal Laser Scanning Microscopy of Monospecies Biofilms in the Presence of NUPs

**FIGURE 3:**
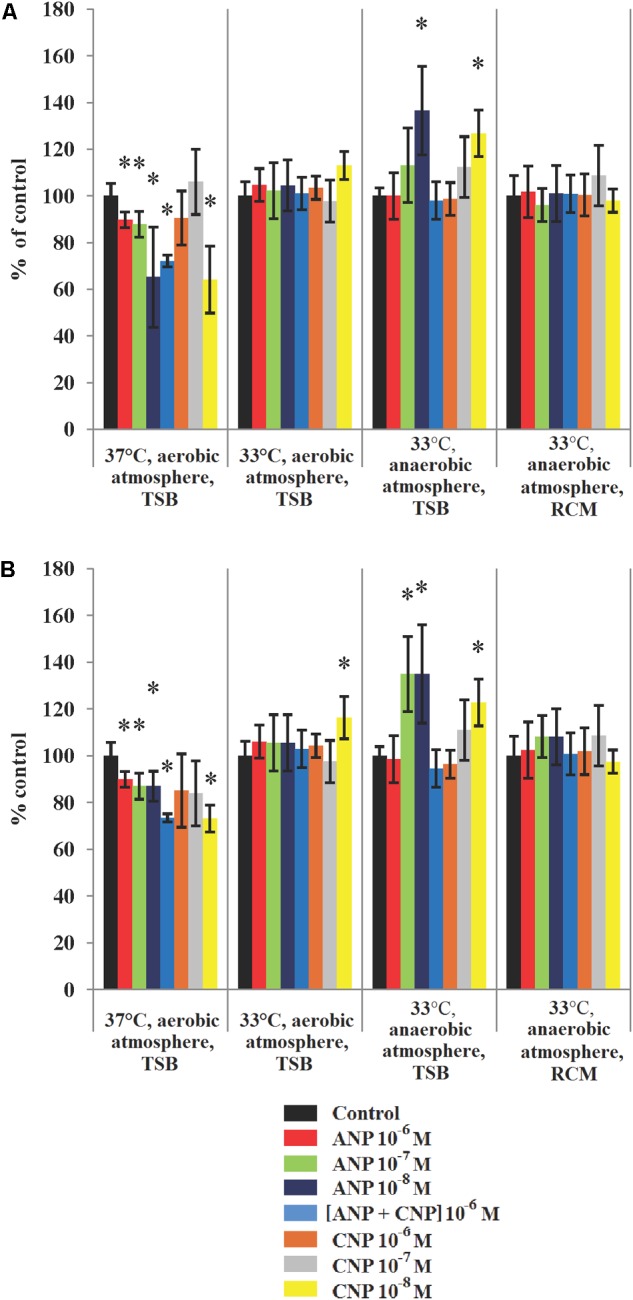
Parameters of monospecies biofilms of *S. aureus* MFP03 measured by CLSM in different cultivation conditions. **(A)** Average biofilm biomass density and **(B)** Average biofilm thickness. ^∗^*P* < 0.05.

Analysis of the mean biofilm thickness and biofilm biomass density of *S. aureus* MFP03 revealed the crucial significance of cultivation conditions in observing the effects of ANP and CNP (Figure 3). It is interesting to note that the biggest impact was observed at low concentrations of both ANP and CNP, i.e., 10^-8^ M. In the absence of treatment and in aerobic conditions at 37°C, the biomass density and the average thickness of the biofilm formed by *S. aureus* MFP03 were 27.66 ± 1.46 μm^3^/μm^2^ and 30.13 ± 1.69 μm, respectively. At all tested concentrations, ANP inhibited *S. aureus* MFP03 biofilm growth. Adding ANP 10^-8^ M decreased the average biomass density of the biofilms to 18 ± 5.9 μm^3^/μm^2^ (Figure 3A) and decreased the average thickness of the biofilms to 25.6 ± 1.9 μm (Figure 3B). CNP significantly inhibited *S. aureus* biofilms only at the lowest concentration (10^-8^ M) where it decreased the biomass density to 17.7 ± 4 μm^3^/μm^2^ and the average biofilm thickness to 22 ± 0.5 μm. The association of the two NUPs (ANP and CNP; 10^-6^ M each) had a slight synergistic inhibitory effect on biofilm parameters. Individually, ANP 10^-6^ M decreased the average biomass density and thickness of the biofilms to 24.8 ± 0.9 μm^3^/μm^2^) and 27.1 ± 1 μm, respectively, while CNP 10^-6^ M alone reduced these values to 24.9 ± 3.2 μm^3^/μm^2^ and 25.8 ± 4.7 μm, respectively. Combining the two NUPs decreased the biomass density and thickness to 19.9 ± 0.7 μm^3^/μm^2^ and to 22.11 ± 0.5 μm, respectively. At 33°C in aerobic conditions, no effect of the peptides was observed except in terms of mean thickness, where an increase was observed in the presence of CNP 10^-8^ M. More precisely, at 33°C in aerobic conditions the average biomass density was only 14.07 ± 0.85 μm^3^/μm^2^, and the average biofilm thickness fell to 14.1 ± 0.9 μm. Then, at 33°C and in aerobic conditions, *S. aureus* MFP03 showed reduced biofilm formation activity in comparison to that observed aerobically at 37°C. When ANP or CNP were added, the biofilm biomass density varied from 14.21 ± 1 μm^3^/μm^2^ to 15.9 ± 0.84 μm^3^/μm^2^ after exposure to CNP 10^-8^ M. The average thickness varied from 13.74 ± 1.3 μm to 16.3 ± 1.3 μm with CNP 10^-8^ M. Then, at 33°C the weak inhibitory effect of both NUPs observed at 37°C disappeared and a tendency to stimulate emerged.

When *S. aureus* was grown in anaerobic conditions at 33°C, we observed that the biofilms reached a biomass density of 16.7 ± 0.58 μm^3^/μm^2^ and an average thickness of 17.3 ± 0.66 μm. These biofilms appeared denser and thicker than biofilms grown at 33°C in an aerobic atmosphere but remained less developed at 37°C. High concentrations (10^-6^ M) of both peptides did not significantly affect biofilm formation in these growth conditions (anaerobic, 33°C). In contrast, we observed that peptides used at 10^-7^ M and 10^-8^ M stimulated biofilm growth (Figure 3). In the presence of ANP 10^-8^ M, biofilm biomass density reached 22.8 ± 3.1 μm^3^/μm^2^ and an average thickness of 23.2 ± 3.6 μm. In the same conditions, CNP increased the average biomass density and thickness up to 21.1 ± 1.7 μm^3^/μm^2^ and 21.2 ± 1.7 μm, respectively. At a concentration of 10^-7^ M the effect of CNP on the biofilm density and thickness was more limited. Exposure of *S. aureus* to both peptides in association didn’t bring any advantage and didn’t have any additional effects. Moreover, changing the TSB medium to RCM led to the disappearance of all significant stimulatory effects of NUPs (Figure 3).

**FIGURE 4:**
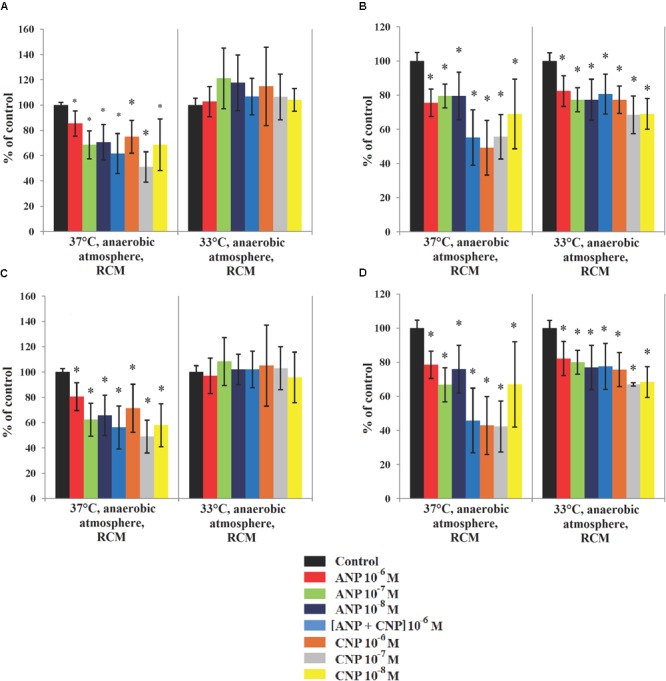
Parameters of monospecies biofilms of *C. acnes* measured by CLSM in different cultivation conditions. **(A)** Average *C. acnes* RT4 biofilm biomass density; **(B)** Average *C. acnes* RT5 biofilm biomass density; **(C)** Average *C. acnes* RT4 biofilm thickness; **(D)** Average *C. acnes* RT5 biofilm thickness. ^∗^*P* < 0.05.

When the same experiment was performed on *C. acnes*, we observed that both strains were sensitive to NUPs. Of course, as RT4 and RT5 strains are unable to grow in the presence of oxygen, the effect of NUPs was only investigated in anaerobic conditions. At 37°C the biofilm formation activity of *C. acnes* RT4 was inhibited by both NUPs alone or in association (Figures 4A,C). In control conditions the biomass density and average thickness of the biofilm formed by C. acnes RT4 were 20.1 ± 0.4 μm^3^/μm^2^ and 23.2 ± 0.5 μm, respectively. ANP maximally reduced RT4 biofilms when 10^-7^ Ì: density and thickness decreased to 12.5 ± 2.6 μm^3^/μm^2^ and 15.9 ± 2.5 μm, respectively. When ANP was used at a concentration of 10^-8^ M we observed the same effect. CNP was more efficient at a concentration of 10^-7^ M, with thickness and density biofilms values that reached only 51 ± 12 and 49 ± 13% of the control, respectively. Combining ANP and CNP had a limited effect on *C. acnes* RT4 biofilm formation. NUPs also exerted an inhibitory effect on the biofilm formation activity of *C. acnes* RT5 when grown at 37°C. In control conditions at 37°C, *C. acnes* RT5 had strong biofilm formation activity (26.9 μm average thickness and 23.3 μm^3^/μm^2^ biomass density) (Figures 4B,D). At all tested concentrations ANP inhibited *C. acnes* RT5 biofilm density and thickness to a level of 67–79% and 75.5–79.5% of the control, respectively. The maximal effect of CNP was noted at 10^-6^ M, with a decrease in biofilm thickness and density to a level of 42.3 ± 17 and 49.2 ± 16% of the control, respectively. CNP 10^-7^ M had virtually the same effect. In contrast, combining ANP and CNP had no synergistic effect.

When the incubation temperature of the two *C. acnes* strains was reduced to 33°C, their biofilm formation activity and sensitivity to ANP and CNP differed. The RT4 strain produced thinner biofilms of about 8.9 ± 0.49 μm^3^/μm^2^ biomass density and 9.3 ± 0.5 μm thickness. Moreover, no inhibitory effect of ANP and CNP was observed and even a marginal stimulation of the biomass density was noted (Figures 4A,C), especially in presence of ANP. Indeed, ANP 10^-7^ M increased RT4 strain biofilm biomass density and average thickness to 121.1 ± 24 and 108.3 ± 19% of the control. The biofilm formation activity of *C. acnes* RT5 at 33°C was also decreased but the effect was more marginal than that of the RT4 strain, with an average thickness and biomass density of 20.7 ± 0.9 μm^3^/μm^2^ and 21 ± 1 μm, respectively (Figures 4B,D). The inhibitory effect of ANP and CNP was preserved though it was weaker. The strongest effect of ANP was observed at a concentration of 10^-8^ M with a decrease in biofilm density and thickness to 77 ± 12 and 77 ± 13% of the control, respectively. CNP exerted the strongest inhibitory effect when it was used at a concentration of 10^-7^ M, leading to a biofilms thickness and biomass density of 68.5 ± 11 and 67 ± 1% of the control, respectively. This shows that the effect of NUPs on *C. acnes* is generally inhibitory but is also strain-specific and can be modulated by its microenvironment. This can be correlated to the behavior and ecological role of *C. acnes* strains in different human skin niches. On the other hand, the general lack of additive or synergistic effect of ANP and CNP suggests that the two peptides have competing effects on bacteria.

### Interaction of *S. aureus* MFP03 and *C. acnes* in Mixed Biofilms

First, we analyzed how the co-existence of *S. aureus* MFP03 and *C. acnes* strains in mixed biofilms produced on glass fiber filters can affect their growth at 37°C. We observed that in biofilms, *C. acnes* forms very solid microcolonies, and these aggregates were impossible to disperse as single cells even by sonication or enzymatic treatment (data not shown). Therefore, we decided to measure cultivable bacteria present in the biofilm by direct counting of *S. aureus* CFU from dispersed biofilms and to determine the metabolic activity present in the biofilms by MTT staining, allowing us to estimate indirectly the evolution of the *C. acnes* biomass.

**FIGURE 5:**
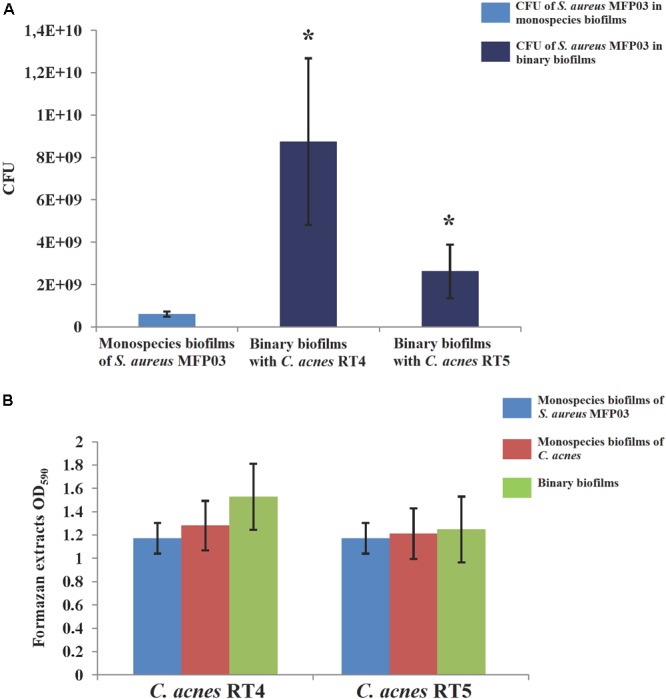
Parameters of mixed biofilms of *S. aureus* MFP03 and *C. acnes* strains at 37°C. **(A)** *S. aureus* MFP03 CFU and **(B)** MTT biofilm values. ^∗^*P* < 0.05.

An average amount of 3.2 × 10^7^ ± 4.1 × 10^6^ *S. aureus* CFU was distributed on the filters. After 72 h of culture at 37°C in monospecies biofilms, the number of cultivable *S. aureus* MFP03 recovered from the monospecies biofilms reached a mean of 6.1 × 10^8^ ± 1.2 × 10^8^ CFU. In the presence of *C. acnes, S. aureus* grew more effectively. More precisely, using the *C. acnes* RT4 strain or the *C. acnes* RT5 strain (8.75 × 10^9^ ± 3.9 × 10^9^ CFU) in co-cultures, the amount of *S. aureus* MFP03 CFU recovered in the presence of the RT4 and RT5 strains of *C. acnes* was 2.62 × 10^9^± 1.3 × 10^9^ CFU and 8.75 × 10^9^± 3.9 × 10^9^, respectively (Figure 5A). The MTT staining of biofilms showed that the average staining intensity of monospecies *S. aureus* MFP03 and *C. acnes* biofilms were virtually equal (Figure 5B). However, it is interesting to note that in the case of mixed biofilms, the MTT values were far more than the values for the sum of monospecies biofilms. In addition, the CFU counts showed that *S. aureus* grew better in mixed biofilms. These results suggest that the growth of *C. acnes* was reduced in the presence of *S. aureus* MFP03.

To simulate better the skin microenvironment where *C. acnes* and *S. aureus* can coexist, we decided to study mixed biofilms at 33°C as the skin surface is not at 37°C and much closer to 33°C (Ariyaratnam and Rood, 1990; Boutcher et al., 1995). Also, to analyze and model the probable effects of colonization, we studied different types of mixed biofilms. We used only strain RT5 because in experiments with CLSM of monospecies biofilms, it demonstrated an inhibitory effect when exposed to ANP and CNP at 33°C, whereas *S. aureus* MFP03 was insensitive to NUPs at this temperature. We observed that in the case of simultaneously grown mixed biofilms and in mixed biofilms for which pre-formed *S. aureus* MFP03 biofilm was first formed, there was no significant difference between the CFU amount of *S. aureus* MFP03 in monospecies and mixed biofilms. In the case of mixed pre-formed *C. acnes* biofilms, the amount of *S. aureus* MFP03 CFU recovered from monospecies biofilms was higher compared to mixed biofilms (7.6 × 10^9^ ± 3.8 × 10^8^ and 4.6 × 10^9^± 3 × 10^8^ CFU, respectively). Likewise, in the case of *C. acnes* biofilms, the amount of *S. aureus* CFU in monospecies biofilms was higher compared to other binary biofilms studied: 7.6 × 10^9^± 3.8 × 10^8^ compared to 4.8 × 10^9^± 1.3 × 10^9^, 4.5 × 10^9^± 7.2 × 10^8^, and 4.2 × 10^9^± 6.8 × 10^8^ CFU for simultaneously grown biofilms in aerobic and anaerobically grown *S. aureus* biofilms, respectively. That should be explained by the cell death level in long-term incubated monospecies *S. aureus* MFP03 biofilms. Indeed, in the case of simultaneously grown biofilms, both monospecies and mixed biofilms were incubated for 3 days, whereas in the case of pre-formed biofilms *S. aureus* MFP03 biofilms needed to be grown for 4 days (with a replacing of filters with biofilms on a fresh medium).

The MTT staining of monospecies and mixed biofilms at 33°C showed that for each type of mixed biofilm, the MTT staining value was less than the sum of the intensities obtained independently for each monospecies biofilm, suggesting that *C. acnes* RT5 grew less in mixed biofilms than in monospecies biofilms. Nevertheless, this inhibitory effect was not as pronounced as in studies realized at 37°C, especially in the case of preformed *C. acnes* RT5 biofilms. More precisely, the OD_595_ of formazan extracts in that case were 1.92 ± 0.04 and 1.7 ± 0.21 for monospecies *S. aureus* MFP03 and *C. acnes* RT5 biofilms, respectively, but reached 2.95 ± 0.035 for the binary biofilms, i.e., 81.6% of the algebraic sum of the two monospecies biofilms. These data suggest that *C. acnes* in mature biofilms can tolerate the negative effects of *S. aureus* MFP03, and that mature biofilms of *C. acnes* are not favorable for *S. aureus* MFP03 adhesion.

### Effects of ANP and CNP on Mixed Biofilms Formed by *C. acnes* RT5 and *S. aureus* MFP03

To investigate the potential effects of NUPs on mixed biofilms we used liquid RCM and grew biofilms simultaneously in equilibrium with planktonic cultures. We decided to test concentrations of the NUPs that had the strongest effect on monospecies biofilms in experiments with CLSM, i.e., 10^-8^ M for ANP and 10^-7^ M for CNP.

**FIGURE 6:**
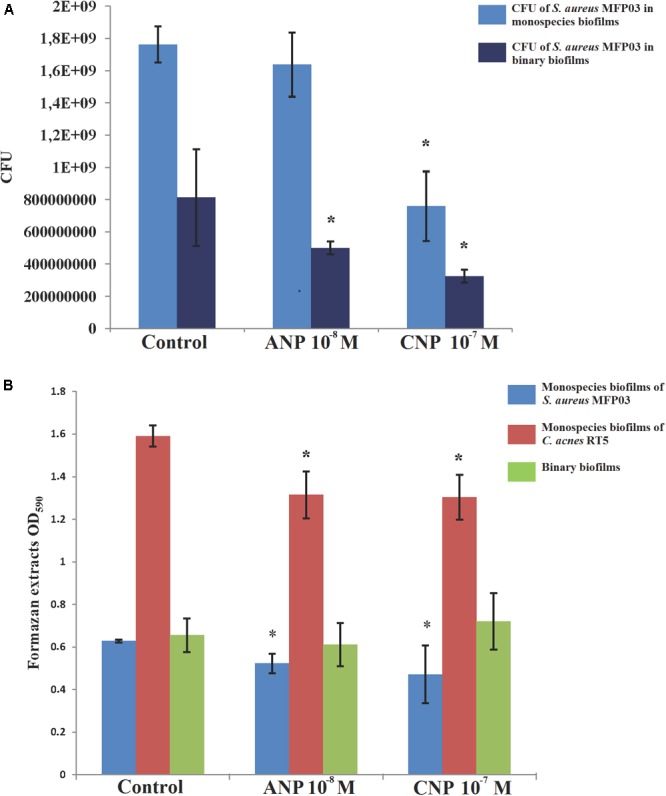
Effects of ANP and CNP on mixed biofilms of *S. aureus* MFP03 and *C. acnes* RT5 at 33°C. **(A)** *S. aureus* MFP03 CFU and **(B)** MTT biofilm values. ^∗^*P* <0.05.

In the presence of NUPs, the number of *S. aureus* MFP03 CFU obtained from monospecies biofilms was decreased (Figure 6A) ranging from 1.76 × 10^9^± 1.1 × 10^8^ CFU in control studies to 1.64 × 10^9^± 2 × 10^8^ CFU with ANP and only 7.58 × 10^8^± 2.2 × 10^8^ CFU with CNP. This suggests that, if cells must adhere to the surface from a planktonic state in the presence of the peptides, NUPs should interfere with this process. This could be an explanation for the decrease in cultivable bacteria obtained from experiments in static conditions on filters, since biofilm production for CLSM experiments cells requires an initial adhesion step.

In mixed biofilms, the number of *S. aureus* MFP03 CFU measured was 2.15-fold lower than monospecies biofilms (8.1 × 10^8^± 3 × 10^8^ CFU, Figure 6A). Adding ANP further reduced *S. aureus* development in mixed biofilms (3.3-fold reduction with an average of 5 × 10^8^± 4 × 10^7^ CFU recovered from the biofilms). In contrast, adding CNP did not significantly change the ratio between planktonic and biofilm bacteria in monospecies and binary biofilms although in binary biofilms the number of *S. aureus* MFP03 was 2.33-fold lower than in mixed ones (3.25 × 10^8^± 4.1 × 10^7^ CFU).

It is interesting to note that the *S. aureus* CFU count results were correlated to MTT values (Figure 6B). MTT staining, corresponding to the metabolic activity in biofilms, was reduced in mixed biofilms in comparison to *S. aureus* MFP03 monospecies biofilms grown in the presence of ANP and CNP (-17.4 and -25.4%). The same was observed with *C. acnes* RT5, where an 18.7% reduction of metabolic activity was observed between monospecies and binary biofilms in the presence of ANP or CNP. However, the more striking result was that the MTT value of mixed biofilms was of the same range in control studies (OD = 0.65 ± 0.08) and in the presence of ANP (0.61 ± 0.1) or CNP (0.72 ± 0.13). These data suggest that *S. aureus* MFP03 is the dominant component of dual-species biofilms but, more importantly, considering the absence of variation of MTT values in binary biofilms and the decrease of cultivable *S. aureus* MFP03, that the balance shifted to *C. acnes* RT5 in presence of NUPs.

Considering these results and the hypothesis, we decided to go further by investigating by confocal microscopy-mixed biofilms labeled with a FISH probe for *C. acnes*. In control studies, we exposed monospecies biofilms of *S. aureus* with this probe for *C. acnes* and we observed no false hybridization. Then, we calculated the percentage of hybridization for each repeat in mixed biofilms and, in parallel, we stained with the FISH-probe monospecies *C. acnes* RT5 biofilms (data not shown). Coefficients varied from 0.2 to 0.99. DAPI staining was used as an indicator of total biomass in binary biofilms. These values were used to calculate *C. acnes* biomass in mixed biofilms. NUPs were tested at the same concentration as described above.

**FIGURE 7:**
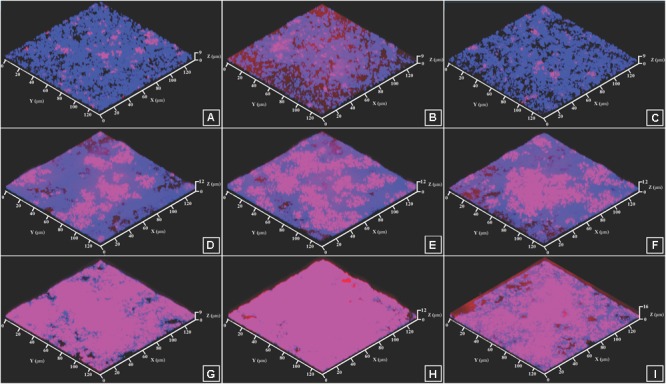
CLSM images of FISH-labeled mixed biofilms of *S. aureus* MFP03 and *C. acnes* RT5. **(A)** Simultaneously grown biofilm, Control; **(B)** Simultaneously grown biofilm, ANP 10^-8^ M; **(C)** Simultaneously grown biofilm, CNP 10^-7^ M; **(D)** Biofilm based on pre-formed *S. aureus* MFP03 biofilm, Control; **(E)** Biofilm based on pre-formed *S. aureus* MFP03 biofilm, ANP 10^-8^ M; **(F)** Biofilm based on pre-formed *S. aureus* MFP03 biofilm, CNP 10^-7^ M; **(G)** Biofilm based on pre-formed *C. acnes* RT5 biofilm, Control; **(H)** Biofilm based on pre-formed *C. acnes* RT5 biofilm, ANP 10^-8^ M; **(I)** Biofilm based on pre-formed *C. acnes* RT5 biofilm, CNP 10^-7^ M. Pink, *C. acnes* RT5 FISH labeling; Blue, DAPI staining (total biomass).

**FIGURE 8:**
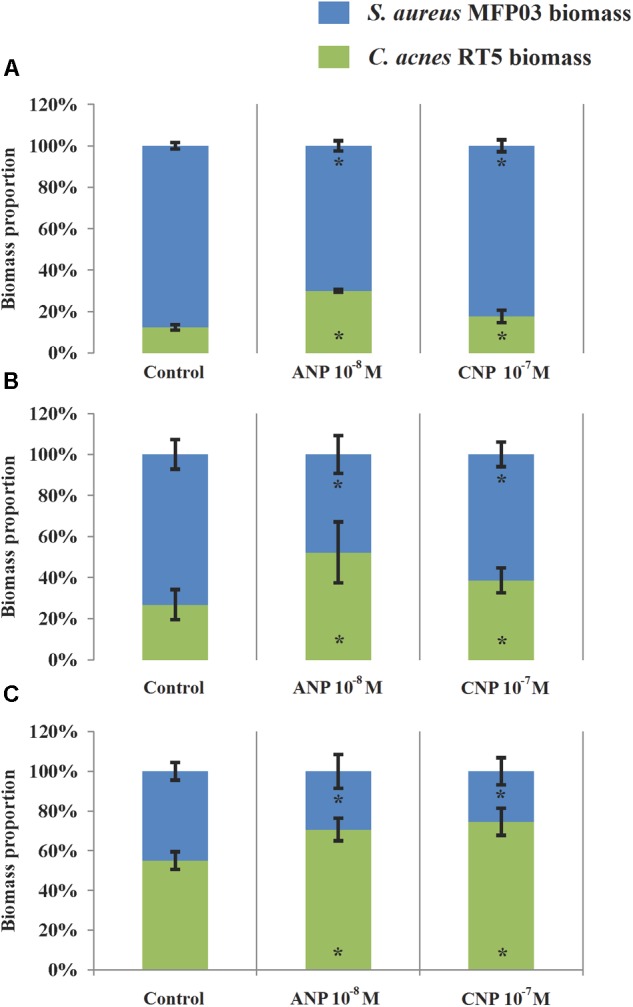
Contribution of *C. acnes* RT5 and *S. aureus* MFP03 to binary biofilms formed in the absence or presence of ANP or CNP. **(A)** Simultaneously grown biofilms; **(B)** Biofilms based on pre-formed *S. aureus* MFP03 biofilms; **(C)** Biofilms, based on pre-formed *C. acnes* RT5. ^∗^*P* < 0.05.

Using simultaneously formed biofilms, in control studies the DAPI labeling illustrating the total amount of bacteria was 2.93 ± 1.2 μm^3^/μm^2^. In the presence of ANP and CNP it was 2.87 ± 1.6 and 3.06 ± 1.1 μm^3^/μm^2^, respectively, showing no significant change in total biomass density (Figure 7). However, in all types of mixed biofilms, adding ANP or CNP increased *C. acnes* RT5 biomass. Indeed, the biomass of *C. acnes* RT5 rose from 0.36 ± 0.18 μm^3^/μm^2^ in the control studies to 0.85 ± 0.53 and 0.54 ± 0.28 μm^3^/μm^2^ in the presence of ANP and CNP, i.e., +236 and +150%, respectively. Taking into account *C. acnes* biomass in monospecies biofilms, these results demonstrated that the proportion of *C. acnes* RT5 in binary biofilms in the presence of ANP and CNP increased to 31 and 18%, respectively, whereas it was only of 12% in the control biofilms (Figures 7A–C, 8A). These results are coherent with CFU counts and MTT measurements.

As noted in simultaneously formed biofilms, using binary biofilms grown in *S. aureus* MFP03 pre-formed biofilms, we observed that ANP increased *C. acnes* RT5 biomass. In control studies it was an increase of 2.9 ± 1.1 μm^3^/μm^2^ (Figure 7D) and reached 3.1 ± 1.6 μm^3^/μm^2^ after exposure to ANP (Figure 7E). Conversely, the biomass of *C. acnes* measured after exposure to CNP decreased to 0.8 ± 0.4 μm^3^/μm^2^ (Figure 7F). This stark reduction of *C. acnes* biomass in the presence of CNP can be attributed to the strong reduction of the *S. aureus* MFP03 CFU count previously observed, suggesting that in these conditions CNP should exert a strong inhibitory effect on biofilm formation. However, it is interesting to note that the calculated relative percentage of *C. acnes* produced on pre-formed *S. aureus* biofilms evolved from 21% in control studies to 40.3 and 36.3% in the presence of ANP and CNP (Figure 8B). This suggests that *S. aureus* biofilm is favorable for *C. acnes* adhesion and that the decrease in *C. acnes* biomass in biofilms observed with CNP results from the strong reduction of *S. aureus* development provoked by this NUP.

This hypothesis is coherent with results from studies realized on mixed biofilms based on pre-formed *C. acnes* RT5 biofilms showing and increase in total biofilm biomass from 2.7 ± 1.3 to 2.9 ± 0.5 and 3.4 ± 0.7 μm^3^/μm^2^ under the effect of ANP and CNP, respectively. This increase can be essentially attributed to a rise of the specific *C. acnes* RT5 biomass, which evolved from 55% in control studies to 68.2 and 74.5% after exposure to ANP and CNP, respectively (Figures 7G–I, 8C). Although, particularly in the case of *S. aureus*, the differences in growth kinetics of the two bacterial species could impact their survival in studies requiring long-term cultivation, these results suggest that the surface of *C. acnes* biofilms cannot be favorable for *S. aureus* adhesion and development.

## Discussion

Natriuretic peptides, which are synthesized and released by cardiomyocytes (ANP) and endothelium cells of capillary vessels (CNP), reach different zones of the organism through the bloodstream and by local diffusion, where temperature and oxygen concentrations are quite different. The skin is one of these organs, characterized by important local variations of the microenvironment. Indeed, whereas in surface and at the level of the stratum corneum the percentage of oxygen is that of the atmosphere (21%), it falls to 3% at the base of the stratum spinosum (Grillon et al., 2012), leading to microaerophilic and even probably anaerobic conditions in specific niches. Similarly, the abundance of anastomoses in the skin allows for important changes in microcirculation and therefore of the local temperature. In particular, in forehead skin, a site where acne appears very frequently, the mean temperature is close to 33°C (Ariyaratnam and Rood, 1990; Boutcher et al., 1995). The hair follicle and sebaceous gland, from which acne generally emerges, is then exposed to very specific environmental conditions in addition to high local concentrations of neuroendocrine factors, such as NUPs, that can be released by the dense capillary network present at the level of the bulge (Xiao et al., 2013). Because of the very small distance between capillary vessels and the skin’s microbial community in that zone, lipophilic bacteria such as *C. acnes* and *S. aureus* are therefore exposed in permanence to NUPs.

*Cutibacterium acnes* is one of predominant components of the skin microbiome (Petersson et al., 2018). It is suspected to be involved in acne formation, but its role remains unclear (Tomida et al., 2013). Indeed, inside skin glands and hair follicles live hundreds of microbial species; thus, it is premature to designate *C. acnes* as the only causative agent of acne. Recent studies have shown that *S. aureus* can be numerous inside inflammatory acne ulcers (Totté et al., 2016; Dreno et al., 2017). To date there is no evidence that *S. aureus* can be a causative agent of acne vulgaris, but its role in acne formation is expected. In addition, we can mention that *S. aureus* colonizes many skin areas affected by psoriasis and atopic dermatitis (Elfatoiki et al., 2016; Lacey et al., 2016), and causes inflammation and treatment complications. Inside skin glands and hair follicles, the quite aerotolerant *C. acnes* prefers to colonize anaerobic micro-niches (Matard et al., 2013), where it forms complex biofilms that include *S. aureus*.

We have recently shown that NUPs can affect significantly *S. epidermidis* and *S. aureus* biofilm formation activity (Gannesen et al., 2018b). Now we show the effect of ANP and CNP in the regulation of monospecies and mixed biofilms of *C. acnes* and *S. aureus* and explored for the first time the influence of oxygen concentrations and temperature. The effect of NUPs on *S. aureus* and *C. acnes* is supported by the observation that the N-acetylmuramoyl-L-alanyl amidase of *C. acnes* (NCBI reference sequence WP_042852295.1) presents 69% homology with the *P. aeruginosa* PAO1 AmiC previously identified as the CNP receptor in this bacterial species (Rosay et al., 2015). In the case of *S. aureus*, no significant homology with the human NUP receptor was found in the sequenced genomes, but we noted that another amidase of Propionibacteriaceae (NCBI reference sequence WP_002530699.1) shows 50% homology with the GatA subunit of the aspartate-tRNA/glutamate-tRNA-amidotransferase of *S. aureus* (NCBI reference sequence WP_072460603.1), suggesting that this protein could also act as a NUP sensor.

Natriuretic peptides appear deeply involved in the interactions between the human skin and its microbiome. NUPs affect mostly biofilms and less planktonic cultures, which could be explained by the fact that, in the humans, microbes live predominantly in multispecies biofilms. This is particularly specific to the skin, where physico-chemical conditions do not allow bacteria to grow in planktonic form. In the present study, we demonstrate that NUPs have not only many different functions as human neurohormones, but also that NUPs are able to affect the commensal skin human microbiota.

Differences in effects, and their dependence on concentration for different microorganisms, could be an evolutionary adaptation of the human hormone system for interacting with microbiota. At first view illogical effects, independent of NUPs concentrations, were observed, particularly in the case of *S. aureus* MFP03. However, NUPs are known to form aggregates at high concentrations (Torricelli et al., 2004) that could limit their activity and make it less efficient than at low doses. In addition, *S. aureus* mainly prefers to colonize the surface of moist skin areas like the nostrils (Estes, 2011; Feuerstein et al., 2017). Then, *S. aureus* should be generally more remote from capillaries than *C. acnes* and, therefore, inhabits areas where the NUP concentrations should be lower and, in this way, more efficient on this bacterial species.

When we investigated *C. acnes* monospecies biofilms we observed that the action of ANP and CNP was strain specific, suggesting that, as proposed for *S. aureus*, the sensitivity of the different *C. acnes* strains to NUPs could be related to their original microenvironment in skin. Whereas *C. acnes* RT5 was sensitive to NUPs at 37 and 33°C, *C. acnes* RT4 became non-sensitive at 33°C. This could reflect different roles and involvements in acne formation of the *C. acnes* strains as well as differences in human skin adaptation, although both RT4 and RT5 are considered to be acneic strains. We focused our work on the *C. acnes* RT5 strain because in monospecies biofilms it showed a biofilm formation inhibitory response to ANP and CNP at 33°C, suggesting a complex adaptation to the physiology of human skin and longtime close interactions between this strain and the human hormone system.

Then we constructed binary systems where *S. aureus* MFP03 and *C. acnes* RT5 were sensible to NUPs to analyze the interrelations of the two bacteria in the same biofilm and the potential interference with NUPs. First, we studied the growth of bacteria on glass fiber filters on Petri dishes, then in the absence of equilibrium with a planktonic culture in a liquid compartment. In these conditions, binary systems of *C. acnes* RT5 and *S. aureus* MFP03 grew better when the two bacteria were cultivated together than in monospecies biofilms. However, an analysis of MTT values showed that the metabolic activity of mixed biofilms was lower than the sum of monospecies biofilms. This could result from the lower growth kinetics of *C. acnes* in comparison to *S. aureus* in mixed biofilms, making *C. acnes* a weak competitor in the face of *S. aureus*. However, when *C. acnes* RT5 was allowed to form a biofilm before interaction with *S. aureus, C. acnes* RT5 resisted *S. aureus* very well, and the growth of *S. aureus* was partly impaired. Nevertheless, the presence of *C. acnes* RT5 generally affected neutrally or positively the growth of *S. aureus* MFP03.

The effect of NUPs on binary biofilms was studied in another system where biofilms were grown in equilibrium with planktonic cells. This model was selected because it was simpler to work with NUPs and because of the high cost of the peptides that required the use of limited volumes. In this system the growth of *S. aureus* MFP03 was reduced in the presence of *C. acnes* RT5 in comparison to monospecies biofilms despite its higher growth rate. This reveals the dependence on adhesion and the importance of interactions between microorganisms on the very early biofilm formation steps. When the cells were forced to adhere on filters on Petri dishes, *S. aureus* MFP03 had great advantage because of its growth speed. Conversely, when *S. aureus* had to adhere to fibers in the presence of *C. acnes* its growth was reduced, indicating that *C. acnes* RT5 was able to interrupt the adhesion of *S. aureus* MFP03 to glass fibers. Monospecies biofilms of both microorganisms were inhibited in this system in the presence of both NUPs, but in the binary biofilm *C. acnes* RT5 grew better in the presence of both NUPs, suggesting that NUPs could increase the competitive advantages of *C. acnes* RT5. Considering that at 33°C *C. acnes* RT4 became non-sensitive to NUPs, these data suggest that NUPs can exert a highly specific and temperature-dependent control on some acneic strains of *C. acnes*. This confirms that *C. acnes*’ metabolism is temperature-dependent, but the mechanism involved in this adaptation process remains unidentified.

These results lead to the proposition of a functional hypothesis concerning the potential role of NUPs in the skin microbiota. Hence, in the forehead skin, where *C. acnes* and *S. aureus* are present in abundance, in normal healthy conditions the temperature is about 33°C and NUPs increase the competitive advantages of *C. acnes* while they inhibit the growth of *S. aureus*, which is considered more pathogenic and potentially troublesome than *C. acnes* (Brown et al., 2013). In circumstances favorable for *C. acnes* overgrowth (skin pores plugging, comedone formation, etc.), induction of an inflammatory reaction increases the local temperature to 37°C (Casscells et al., 1996; Chanmugam et al., 2017). This increase of temperature should stimulate intensive growth of *S. aureus* and, to a lesser extent, that of *C. acnes*, with a risk of a runaway phenomenon. However, at 37°C NUPs should start to inhibit the growth of both bacteria and thus act as a thermostat to help human defense systems to reduce inflammation. By the same way, this temperature-dependent effect of ANP and CNP could be involved in pathologies such as psoriasis, characterized by wavelike progress (Ayala-Fontánez et al., 2016) and *S. aureus* overgrowth (Totté et al., 2016). Indeed, is appears that in psoriatic plaques, *S. aureus* have strong advantages against other microbial species because of unknown reasons, and because of this it probably forms virtually monospecies biofilms. In remission phases, when the temperature of the skin is about 33°C, *S. aureus* should slowly form biofilms which can cause inflammation and a local temperature increase. While inflammation progresses, with the rise of the temperature NUPs should start to inhibit *S. aureus* biofilm formation. This hypothesis is supported by the fact that most effective concentrations of ANP and CNP active on *S. aureus* are low, and *S. aureus* colonizes sites where the NUP concentration should be low. However, during psoriasis, an exuberant angiogenesis occurs, and by this way the concentrations of NUPs should increase and their inhibitory effect on *S. aureus* should vanish. That could be explained at least in part by the fact that *S. aureus* is so difficult to eradicate in psoriatic lesions. Of course, these hypotheses remain speculative and deserve further clinical investigations.

## Conclusion

More than providing a further demonstration of the diversity and complexity of skin–bacteria communication, this work provides for the first time a potential functional role of ANP and CNP as natural thermostats, allowing the organism to regulate bacterial biofilm development and balance between *C. acnes* and *S. aureus* as a function of the local temperature.

## Author Contributions

AG performed the experiments, analyzed the data, and wrote the draft of the manuscript. OL supervised the work and provided the scientific assistance. P-JR and MB provided the scientific and technical assistance. AN and VP supervised the work. MF headed the funding organization, supervised the work, and assisted with manuscript writing. All authors read and approved the final manuscript.

## Conflict of Interest Statement

The authors declare that the research was conducted in the absence of any commercial or financial relationships that could be construed as a potential conflict of interest.

## Footnotes

1 https://blast.ncbi.nlm.nih.gov/Blast.cgi?PAGE=Proteins
